# Impact of confinement housing on study end-points in the calf model of cryptosporidiosis

**DOI:** 10.1371/journal.pntd.0006295

**Published:** 2018-04-25

**Authors:** Geneva Graef, Natalie J. Hurst, Lance Kidder, Tracy L. Sy, Laura B. Goodman, Whitney D. Preston, Samuel L. M. Arnold, Jennifer A. Zambriski

**Affiliations:** 1 Paul G. Allen School for Global Animal Health, College of Veterinary Medicine, Washington State University, Pullman, Washington, United States of America; 2 Department of Population Medicine and Diagnostic Sciences, College of Veterinary Medicine, Cornell University, Ithaca, New York, United States of America; 3 Department of Allergy and Infectious Disease, School of Medicine, University of Washington, Seattle, Washington, United States of America; University of Washington, UNITED STATES

## Abstract

**Background:**

Diarrhea is the second leading cause of death in children < 5 years globally and the parasite genus *Cryptosporidium* is a leading cause of that diarrhea. The global disease burden attributable to cryptosporidiosis is substantial and the only approved chemotherapeutic, nitazoxanide, has poor efficacy in HIV positive children. Chemotherapeutic development is dependent on the calf model of cryptosporidiosis, which is the best approximation of human disease. However, the model is not consistently applied across research studies. Data collection commonly occurs using two different methods: Complete Fecal Collection (CFC), which requires use of confinement housing, and Interval Collection (IC), which permits use of box stalls. CFC mimics human challenge model methodology but it is unknown if confinement housing impacts study end-points and if data gathered via this method is suitable for generalization to human populations.

**Methods:**

Using a modified crossover study design we compared CFC and IC and evaluated the impact of housing on study end-points. At birth, calves were randomly assigned to confinement (n = 14) or box stall housing (n = 9), or were challenged with 5 x 10^7^ *C*. *parvum* oocysts, and followed for 10 days. Study end-points included fecal oocyst shedding, severity of diarrhea, degree of dehydration, and plasma cortisol.

**Findings:**

Calves in confinement had no significant differences in mean log oocysts enumerated per gram of fecal dry matter between CFC and IC samples (*P* = 0.6), nor were there diurnal variations in oocyst shedding (*P* = 0.1). Confinement housed calves shed significantly more oocysts (*P* = 0.05), had higher plasma cortisol (*P* = 0.001), and required more supportive care (*P* = 0.0009) than calves in box stalls.

**Conclusion:**

Housing method confounds study end-points in the calf model of cryptosporidiosis. Due to increased stress data collected from calves in confinement housing may not accurately estimate the efficacy of chemotherapeutics targeting *C*. *parvum*.

## Author summary

After respiratory disease, diarrhea is the leading cause of death in children < 5 years worldwide. Recent multicenter studies have identified the parasite genus, *Cryptosporidium* to be one of the most common causes of pediatric diarrhea in resource-poor settings. In industrialized nations the parasite is well controlled through centralized water treatment facilities. Consequently, drug development targeting this neglected tropical disease has been limited. There are currently no vaccines to prevent cryptosporidiosis. Nitazoxanide is the only approved drug targeting *Cryptosporidium*, but it has poor efficacy in HIV positive children. Owing to the recently published multicenter findings there have been renewed *Cryptosporidium* drug development efforts. The calf model of cryptosporidiosis is widely recognized as the best approximation of human disease but it is difficult to execute resulting in varied experimental approaches. Here, we describe the two most common methods of executing the calf model of cryptosporidiosis and investigate how these methods impact study end-points used to inform identification of drug candidates and clinical trials. We find that confinement housing significantly increases plasma cortisol in calves and impacts key study end-points. Data collected via confinement housing may not accurately estimate drug efficacy, underscoring the need for model harmonization. The results of this study should be considered when interpreting chemotherapeutic data derived from the calf model of cryptosporidiosis.

## Introduction

*Cryptosporidium* is a genus of protozoal parasites that infect a wide range of hosts including wild and domestic mammals, amphibians, and reptiles [1]. The most pathogenic species are *C*. *hominis* and *C*. *parvum*, which primarily affect people and cattle, respectively. *C*. *parvum*, which is zoonotic, represents an important public health threat. In resource-poor countries this is of particular concern for children who live in close proximity to cattle, and in resource-rich countries the zoonosis impacts agricultural laborers on commercial dairy farms, where herd prevalence can be as high as 80% [2, 3]. In both human and cattle hosts, disease is characterized by villus atrophy, intestinal crypt inflammation, and malabsorptive, maldigestive diarrhea that may be secretory in nature [4, 5]. In addition, large numbers of infective oocysts are shed in the stool, which impacts environmental parasite loading and propagates fecal-oral transmission [6].

Diarrhea has been identified as a leading cause of death in children < 5 yrs globally [7, 8]. In resource-poor countries, cryptosporidiosis is the second leading cause of diarrhea in children (after rotavirus) and is associated with an increased risk of death in the first 2 yrs of life [7]. Individuals that are immunocompromised due to other etiologies, such as HIV or severe malnutrition, are more likely to develop life-threatening diarrhea [1, 7, 9]. Pediatric cryptosporidiosis has also been associated with cognitive impairment and stunting, regardless of the development of diarrhea [9–11]. Despite the adverse impact on human lives there are no treatments or vaccines that are consistently effective against *Cryptosporidium* [12–16]. Nitazoxanide is the only therapy currently approved for the treatment of cryptosporidiosis in people. It has been demonstrated to have modest efficacy in HIV-seronegative children, but not HIV-seropositive children [16]. Similarly, in veterinary patients there is a dearth of treatment options. Halofuginone is the only drug licensed for use in cattle to control cryptosporidiosis, but it is not an ideal treatment option. It is registered in a few countries, excluding the United States, is only effective when used prophylactically and not therapeutically, has a very narrow margin of safety, and is not cost-effective [17–19]. Given the substantial impact of cryptosporidiosis on child health in resource-poor settings and the lack of options to prevent and treat disease in human and animal populations, there is an urgent need to identify and test new chemotherapeutic agents.

To develop effective chemotherapeutics, repeatable and reliable *in vivo* and *in vitro* models are essential. *Cryptosporidium* is notoriously difficult to culture and study *in vitro*, though advances are being made using hollow fiber technology, and *in vivo* models either fail to achieve comparable severity of clinical illness or are cumbersome to execute [20, 21]. Murine models of cryptosporidiosis are commonly used because they permit induction of infection with *C*. *parvum*. Although *C*. *parvum* is not host-adapted to mice, this model is widely applied because it is tractable. *Cryptosporidium* species do not induce severe clinical illness in mice. This includes *C*. *muris*, which is host-adapted to mice [22]. Studies in the mouse model report weight loss, listlessness, and occasional pasty stool, but do not report fulminant diarrhea [23, 24]. The inability to induce measureable clinical illness is a major limitation of mouse models, because we must be able to measure a response to treatment in order to evaluate chemotherapeutic efficacy. The calf model of cryptosporidiosis is the best approximation of human disease because calves experience natural infection and clinical illness that mirrors human symptomology. However, the model is not consistently applied across research studies, which may impact evaluation of study end-points such as fecal oocyst shedding, severity of diarrhea, and degree of dehydration.

The type of housing system used in calf model studies of cryptosporidiosis is dictated by the desired method of fecal sampling. The two methods most commonly used to collect and evaluate stool from calves experimentally infected with *C*. *parvum* are the Complete Fecal Collection (CFC) method and the Interval Collection (IC) method. For CFC the total amount of stool produced by a calf is collected every 24 hours, blended for homogenization, measured either by weight or by volume, and an aliquot (~ 0.2g) is removed for evaluation [25, 26]. In the interval collection method, a small fecal sample (~10 g) is collected directly from the rectum of the calf every 12 or every 24 hours [14, 27–29]. Historically, *Cryptosporidium* challenge studies conducted in human volunteers have used CFC [30, 31]. For human participants CFC is considered to be the gold-standard for evaluating reduction of diarrhea in response to treatment. Another potential advantage to CFC is that homogenization of the stool sample prior to oocyst enumeration mitigates the risk of variation in oocyst counts associated with intermittent oocyst shedding or diurnal fluctuations. Though there is a report of possible diurnal variation in oocyst excretion in confinement housing, it is not clear whether this would be an issue in box stall housing or in all situations of confinement housing [26].

A major disadvantage to CFC is that it is cumbersome and difficult to execute in animal models. With human volunteers, a variety of sample collection methods exist to permit simple, non-invasive collection of CFC. With neonatal calves collection of CFC is a formidable challenge requiring use of confinement housing that severely restricts calf movement and their ability to engage in natural behaviors, such as grooming. Calves may rise or lay down in sternal or lateral recumbancy, but they cannot turn around or ambulate. The use of this degree of confinement is necessary in order to direct the fecal matter expelled by the calf directly into collection pans. Enlarging the confinement system to let the calf ambulate or to allow sufficient space for the calf to turn around would not permit collection of the entire stool sample, especially at the peak of illness when calves may pass stool 1–3 times per hour. This housing system, originally developed for use in the veal industry, is not ideal for calf welfare and may not be acceptable to Institutional Animal Care and Use Committees, particularly those at European institutions. In contrast to CFC, the use of the IC method allows calves to be housed in box stalls where they can move freely. It also has the added advantage of reduced labor requirements for sample collection and calf husbandry.

In addition to required use of confinement housing, implementation of CFC is expensive to maintain, labor intensive, and age restricted. Calf enrollment cannot exceed 10 days as calves older than 10 days are too large for confinement housing. The “Guide for the Care and Use of Agricultural Animals in Research and Teaching” specifically states that calves can not be placed in confinement after day 10 of life [32]. Thus the window of data collection in confinement housing is limited to 10 days. In addition, this limits study efficiency, as it is difficult to manage large numbers of calves, which typically restricts enrollment to 4–6 animals. There is also a risk that data collected during this period may not be representative. It has been demonstrated that calves are more likely to have higher levels of cortisol in the first 10 days of life, indicating higher susceptibility to stress [33, 34]. However, the impacts of confinement housing on cortisol levels have not been evaluated and the impacts of elevated cortisol on study end-points have also not been assessed.

No studies have been conducted comparing CFC and IC, nor are there studies comparing confinement housing and box stalls in the calf model of cryptosporidiosis, which is a substantial knowledge gap considering how integral this model is to *Cryptosporidium* drug development. It is unknown if the IC method is an acceptable alternative to CFC with respect to relevant study end-points such as diarrheal severity and magnitude of fecal oocyst shedding. It is also unknown if these end-points are impacted by calf confinement. Lastly, there are no studies that compare cortisol levels and other negative health outcomes, such as risk of calf injury and need for supportive care, in association with housing type. Therefore, the objective of this study was to determine if data collected via IC is comparable to data collected via CFC, and if confinement housing impacts the study end-points used to evaluate chemotherapeutic agents targeting *C*. *parvum* in the calf model.

## Materials and methods

### Ethical and regulatory approval

This study was reviewed and approved by the Washington State University Institutional Animal Care and Use Committee (ASAF04679). The study adhered to the guidelines put forth by the Animal Welfare Act and specifically the Animal Welfare Act & Regulations Blue Book for USDA animals.

### Calf enrollment, housing, and husbandry

A total of 25 Holstein bull calves were enrolled contemporaneously on a commercial dairy farm located in Pasco, WA. Calves were enrolled for a period of 25 days. To ensure that calves were not exposed to *Cryptosporidium* and to limit exposure to other microorganisms, all calvings were attended and assisted. The perineum of the dam was cleaned thoroughly with a povidone-iodine scrub, and each calf was delivered using a single-use plastic sheet or a designated wheelbarrow that was thoroughly cleaned and disinfected between each calving. A physical exam was conducted and each calf was weighed. Calves with normal physical exam findings that weighed more than 29.5 kg were enrolled. Upon enrollment, the umbilicus of each calf was dipped in an iodine tincture, and 3 ml of vitamin E and selenium (BoSe, Intervet/Merck Animal Health, Germany) was administered subcutaneously. Calves received 4 L of ≥50 g IgG/L commercial colostrum replacer within 3 hours of birth (Bovine IgG, Colostrum Replacement, Land O’Lakes Inc., St. Paul, MN) via an esophageal tube feeder. Calves were transported to Washington State University in a dedicated trailer bedded with sterile straw. Animals were randomly assigned to box stall (n = 9), confinement housing (n = 14), box stall negative control (n = 1), or confinement housing negative control (n = 1) treatments and housed in a BSL 2 isolation facility. Negative control calves served as sentinels to aid in monitoring for inadvertent transmission of parasite between calves. Inadvertent transmission would result in some calves receiving a larger parasite inoculum than others and could impact severity of clinical illness. Calves in box stalls were housed individually in approximately 12.2 m^2^ (40 ft^2^) of space, bedded in sterile wood shavings, and had a mirror placed at eye-level for environmental enrichment. Calves in the confinement housing group were placed inside of commercially manufactured elevated calf stalls (Wenke Manufacturing, Pender, Nebraska) [25, 26]. To provide environmental enrichment, 2 stalls were placed in each BSL 2 room and calves were faced toward each other at a distance of 0.6 m (2 ft) to prevent cross-contamination. The surface area of each stall was 2.7 m^2^ (8.75 ft^2^) (152.4 cm long by 53.3 cm wide), and each stall was raised 27.9 cm off of the ground (S1 Fig). Stalls were constructed from 1.9 cm (0.75 in) galvanized square framed tubing with an open-grate rubber coated floor to permit feces to fall below the stall into collection bins. In compliance with the “Guide for the Care and Use of Agricultural Animals in Research and Teaching,” on day 10 of life, calves randomized to confinement housing were removed from confinement and placed in box stalls [32]. All calves were followed for 25 days post-infection.

Over the course of enrollment, calves were fed every 12 h via nipple buckets with a commercial, non-medicated milk replacer containing 20% crude protein and 20% fat (MaxiCare, Land O’Lakes, Shoreview, MN). Water was provided ad libitum.

### Oocyst inoculation

Calves were randomly assigned to inoculation (n = 23) or negative control groups (n = 2). Calves in the inoculation group were challenged with a commercially available Iowa laboratory isolate of *Cryptosporidium parvum* within 48–72 h from birth at a dose of 5 × 10^7^ oocysts (Bunch Grass Farms, Deary, ID). Oocysts were administered within 1 month of original isolation and were cleaned for one minute in 0.6% sodium hypochlorite to inactivate possible viruses and bacteria co-purified with the oocysts, then washed four times with phosphate buffered saline to remove the sodium hypochlorite. The oocysts were delivered orally in a 5 mL suspension via the rigid oral portion of an esophageal feeder, followed by approximately 120mL of water to ensure that the entire oocyst suspension was given to the calf. Negative control calves were sham challenged to maintain blinding.

### Fecal sample collection

All calves in confinement housing underwent CFC every 24 h and had an IC sample collected every 12 h. Calves in box stalls had an IC sample collected every 24 h. For IC sampling, up to 10 g of stool was collected directly from the rectum of the calf via digital manipulation with a gloved hand. CFC methods previously described for stool collection were used [25]. Briefly, a fecal collection pan was placed beneath each calf, and urine was diverted to a 12 h adult diaper to prevent contamination of the fecal pans. All stool from the fecal pan was collected every 24 h and blended for homogenization to ensure uniform oocyst distribution. The contents of the blender were then weighed (kg). After homogenization, a 0.2 g aliquot of feces was removed from all IC and CFC samples for enumeration via real-time quantitative Polymerase Chain Reaction (qPCR).

### Fecal sample analysis

Oocysts counts were interpolated by qPCR using serial dilutions of commercially purified *C*. *parvum* oocysts (Waterborne, Inc., New Orleans, LA). Total nucleic acid was extracted from supernatants of 200 mg of fecal sample, oocyst suspension, or negative control homogenized in 400 μl of PBS using a magnetic bead based automated procedure (AM1840, Applied Biosytems, Foster City, CA). An exogenous control (MS2 phage) was added to the lysis buffer to control for extraction efficiency [35]. qPCR for *Cryptosporidium* spp. 18S rRNA was performed using a previously described assay on the Applied Biosystems 7500-FAST plaCFCrm using an inhibitor-optimized master mix (Part 95134, Quantabio, Beverly, MA), 300 nM forward primer, 900 nM reverse primer and 120 nM of probe (labeled with FAM) [36].

For both CFC and IC samples, the count was standardized by the fecal dry weight percentage. Dry weight analysis of fecal samples was obtained by taking a 5–10 g portion of each original fecal sample, drying it at 108 °C for a minimum of 24 h (Squaroid Vaccuum Oven, Labline, Kochi, India), then weighing it directly (Scout Pro SP202, Ohaus Corporation, Pine Brook, NJ) [37].

### Calf health monitoring

The calves were examined and evaluated every 2–12 h. A complete physical exam was conducted every 12 h at feeding time. At the time of physical exam any new injuries or clinical signs were documented, this included abrasions and wounds, as well as swellings, areas of localized pain, and fever. In order to minimize inter-observer bias, standardized and validated rubrics were used as previously described [14, 27, 28, 37]. In addition, all research personnel underwent training to maximize inter-observer agreement. Appetite, fecal consistency, mentation (state of mental activity and responsiveness), and hydration status were each individually evaluated on an ordinal scale of 1–3, where a score of 1 represented normal clinical findings and a score of 3 was consistent with severe clinical illness in the specified category (S1 Table). Qualitative measures of hydration such as skin turgor and degree of enophthalmos were used. Monitoring for enophthalmos was necessary for identifying calves at risk for development of corneal ulcers. As severity of dehydration worsens in calves and the globes of the eyes recede further into the orbit, the eyelids roll inward causing the eyelashes to rest directly on the cornea, abrading the surface. Hydration status was also assessed quantitatively via measurement of urine production every 12 h. To determine urine production without the use of urinary catheters, an adult diaper was weighed, secured over the calf’s penis, and re-weighed to determine the difference (kg).

### Cortisol sampling and analysis

Blood samples were collected via jugular venipuncture on days 2, 4, 5, and 9 post-infection. All samples were collected at 10:00 A.M. Samples were obtained by individuals trained in blood collection using a vacutainer system. For all enrolled calves, to reduce the risk of falsely elevating cortisol levels, efforts were made not to create excessive stress prior to blood collection, e.g., loud noises, changes in lighting, use of excessive restraint, more than 2 venipuncture attempts. Calves in confinement housing were already restrained within the housing system. Additional restraint of these animals was not required and could have resulted in additional stress. Blood was collected from calves in confinement housing by reaching directly through the bars of the housing mechanism. Calves in box stalls were restrained in sternal recumbancy to visualize the jugular vein. Restraint was for less than 120 sec and attempts were made to avoid chasing calves to capture them in the stalls. However, calves in box stalls were generally more energetic and therefore ambulated more frequently prior to blood collection.

Cortisol was extracted from calf plasma by solid phase extraction and quantified using ultra performance liquid chromatography in tandem with mass spectrometry (UPLC-MS/MS). Cortisol and cortisol-D_4_ were purchased from Cerilliant (Round Rock, TX) and stock solutions were separately prepared in 25% methanol at concentrations of 20 μg/mL and 750 ng/mL, respectively. A calibration curve was generated in calf plasma at nominal concentrations of 1, 5, 15, 20, 40, 50, 75, 100, 150, and 200 ng/mL. Quality control (QC) samples were prepared at nominal concentrations of 3, 90, and 175 ng/mL. 150 μL of calibration and QC samples were added to a 96-well plate along with the study samples. Next, 20 μL of the cortisol-D_4_ solution was added to each well followed by addition of 300 μL methanol. Samples were aspirated and then centrifuged at 3600 rcf for 20 minutes. Following centrifugation, 300 μL of the resulting supernatant was removed and placed in a 96 well plate. 900 μL of 4% phosphoric acid was added to each 300 μL aliquot, and the samples were mixed thoroughly. Samples were then placed in an Oasis Prime HLB μElution plate (Waters, Milford, MA) and washed with 2 aliquots of 200 μL 25% methanol. Next, 2 aliquots of 90/10 acetonitrile:methanol were added to each sample, and the resulting eluate was diluted with 25 μL water. 7.5 μL of each sample was injected onto an Acquity UPLC in tandem with a Waters Xevo TQ-S. A Zorbax Eclipse Plus C18 column (Agilent, Santa Clara, CA) heated to 40 °C and mobile phases A: water with 0.1% formic acid and B: acetonitrile with 0.1% formic acid were used with the following gradient: 90%-80% A over the first minute, 80%-10% A from 1 to 3.5 minutes, a further decrease to 5% A until 5 minutes, followed by an increase back to 90% A until 7 minutes. The flow rate of the solvents was 0.5 mL/min. The transitions (*m/z*) 363.2>121.1 and 367.2>121.1 were used to quantify cortisol and cortisol-D_4_, respectively. Endogenous cortisol observed in the calf serum was used to generate the calibration curve, and the amount of naturally occurring cortisol was subtracted from each calibration curve and QC sample. All QC samples were within 15% of their expected values, and the coefficients of variation (CV%) were < 15%.

### Statistical analysis

Descriptive and inferential methods were used. The Shapiro–Wilk test was used to determine if the data was non-Gaussian. Hypothesis tests for normally distributed continuous data were analyzed using the student’s t test or analysis of variance. Categorical variables were tested via chi-square or Fisher’s exact. Non-normal continuous variables were either log-transformed or analyzed via the Wilcoxon Rank Sum test.

The Wilcoxon Rank Sum was used to assess differences in fecal oocyst counts and fecal dry matter percentage between IC and CFC samples for confinement housing calves and between IC samples in confinement housing and box stall calves. The Wilcoxon Rank Sum test was also used to evaluate the differences in plasma cortisol, daily weight gain, daily milk replacer consumption, volume of fluid therapy, and frequency of non-fluid therapy treatments in confinement housing and box stall calves. Chi-square or Fisher’s exact tests were used to describe the association between frequency of injury and housing type. The frequency of injury was counted once at the time of diagnosis. The frequency of non-fluid therapy treatment was counted with every intervention following diagnosis. The frequency of injury and non-fluid therapy treatments were recorded in this manner to enable the ability to track the occurrence of new injuries as well as to assess the amount of researcher effort required to subsequently treat and manage those injuries.

Bivariate analysis was used to evaluate the relationship between log oocysts per gram of fecal dry matter and plasma cortisol. In order to identify the best predictors of total fecal output, simple backward stepwise regression was carried out to achieve the most parsimonious model. Individual explanatory variables with a *P*–value ≤ 0.2 in bivariate analysis were retained. Data were analyzed using JMP Pro 11.0 (SAS Institute Inc., Cary, NC).

## Results

Enrolled calves weighed an average of 42.1 ± 4.9 kg at birth (range 36.8–59.3 kg). The mean serum total protein measurement at 48 h of life was 5.8 ± 0.4 (g/dL) (range 5.1–6.4 g/dL). There were no differences in serum total protein across groups (*P* = 0.7), and no calves experienced failure of passive transfer. All calves challenged with *C*. *parvum* oocysts developed fecal oocyst shedding within 3 days post-infection (PI) (45% at 1 day PI, 41% at 2 days PI, and 14% at 3 days PI). The median duration from parasite challenge to onset of fulminant diarrhea and clinical illness consistent with cryptosporidiosis was 3 days post-infection (3.2 ± 0.4). On day 10 PI, all calves were still shedding oocysts in their stool. Stool was tested via qPCR for co-infection. All enrolled calves were negative for *E*. *coli* K 99, *Salmonella* spp., rotavirus, and corona virus. The two negative control calves did not develop cryptosporidiosis. In order to prevent calves from turning around in the stall and urinating in the fecal collection bin, we applied the head-catch to 2 animals. Both calves died after they became cast in the stall and were unable to rise. These animals were removed from the study. Final analysis included 12 confinement housed calves.

**Fig 1:**
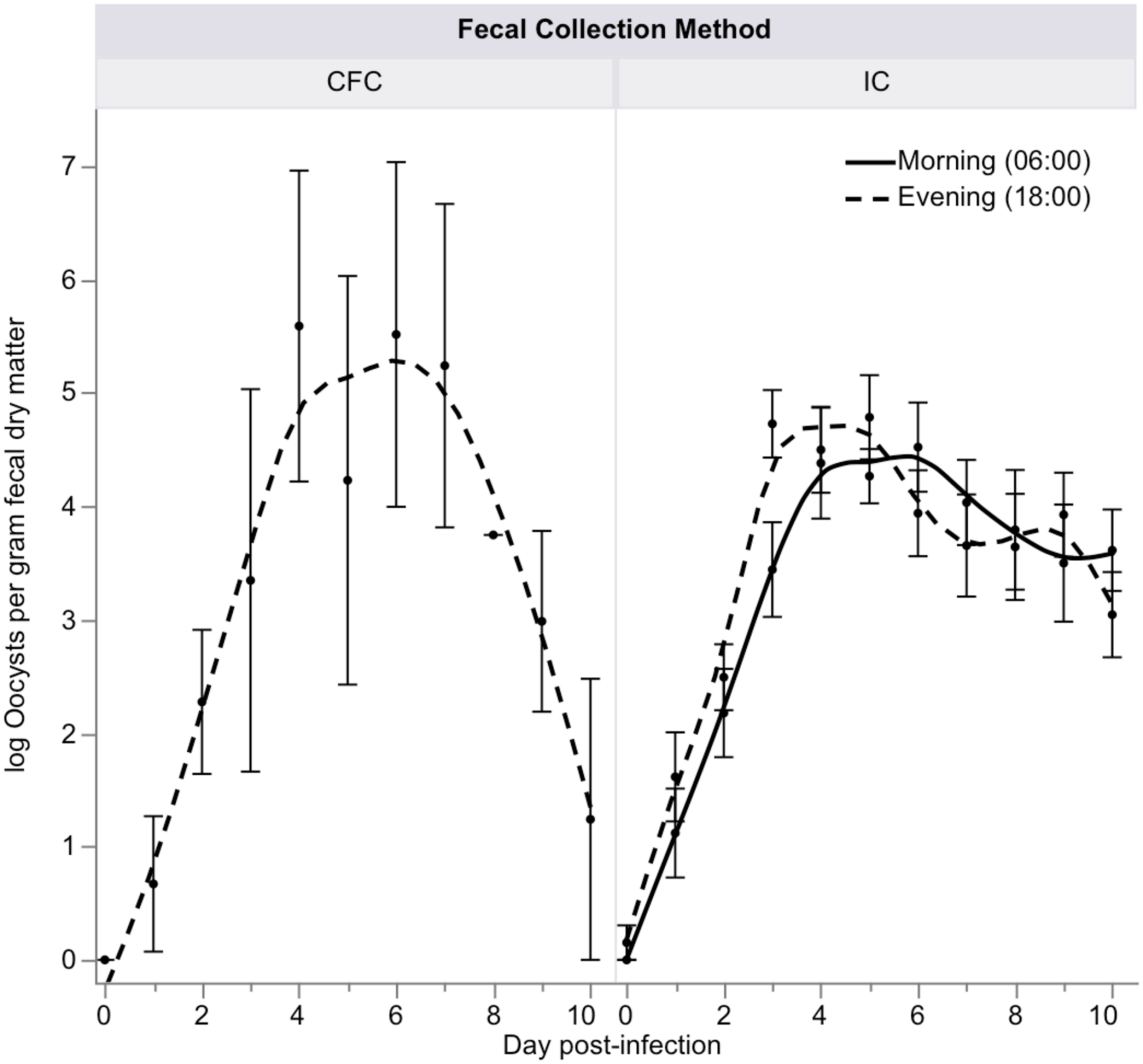
Comparison of fecal oocyst enumeration (mean ± SE) for stool samples collected via interval collection (IC) and complete fecal collection (CFC) from confinement housing calves (n = 12) experimentally infected with *C*. *parvum*. Morning and evening IC samples were compared. CFC samples were evaluated once every 24 hrs in the evening.

For calves housed in confinement housing (n = 12), the mean total fecal output was 4.8 ± 3.8 kg/day (range 0.1–15.7 kg/day) and a mean total urine output was 1.8 ± 1.4 kg/day (range 0.1–5.8 kg/day) during the 10 day study period (Table 1). The uninfected control calf in confinement housing had a mean total fecal output of 0.5 ± 0.6 kg/day (range 0.1–2.1 kg/day) and a total mean urine output of 1.7 ± 1.0 kg/day (range 0.4–3.9 kg/day) (Table 1). Over the 10 day period of confinement, there were no significant differences in mean log oocysts enumerated per gram of fecal dry matter between CFC (10.8 ± 9.8) and IC samples (11.7 ± 9.5) (*P* = 0.6), nor were there any diurnal variations in oocyst shedding for morning and evening IC fecal samples (*P* = 0.1) (Fig 1). The fecal dry matter percent was significantly lower in CFC samples (*P* = 0.01) (Table 1).

**Table 1:** Descriptive summary of daily and stool and urine production for calves (n = 12) in confinement housing from days 0 to 10 post-infection.

Parameter		CFC^a^	IC^b^	
			AM	PM
Stool production/day (kg)	mean ± SD	4.8 ± 3.8	-	-
	IQR	1.2–7.3		
Urine production/day (kg)	mean ± SD	1.8 ± 1.4	-	-
	IQR	0.5–1.9		
Fecal dry matter percentage/day	mean ± SD	9.8 ± 9.5*	12.3 ± 9.8	12.3 ± 9.3
	IQR	4.6–16.1	5.4–18.5	4.5–19.2
Daily log oocysts/g fecal dry matter	mean ± SD	3.4 ± 1.9	3.3 ± 1.8	3.3 ± 1.8
	IQR	2.5–4.1	2.3–4.5	2.1–4.4

* *P*–value < 0.05

^a^ Complete fecal collection

^b^ Interval collection

**Fig 2:**
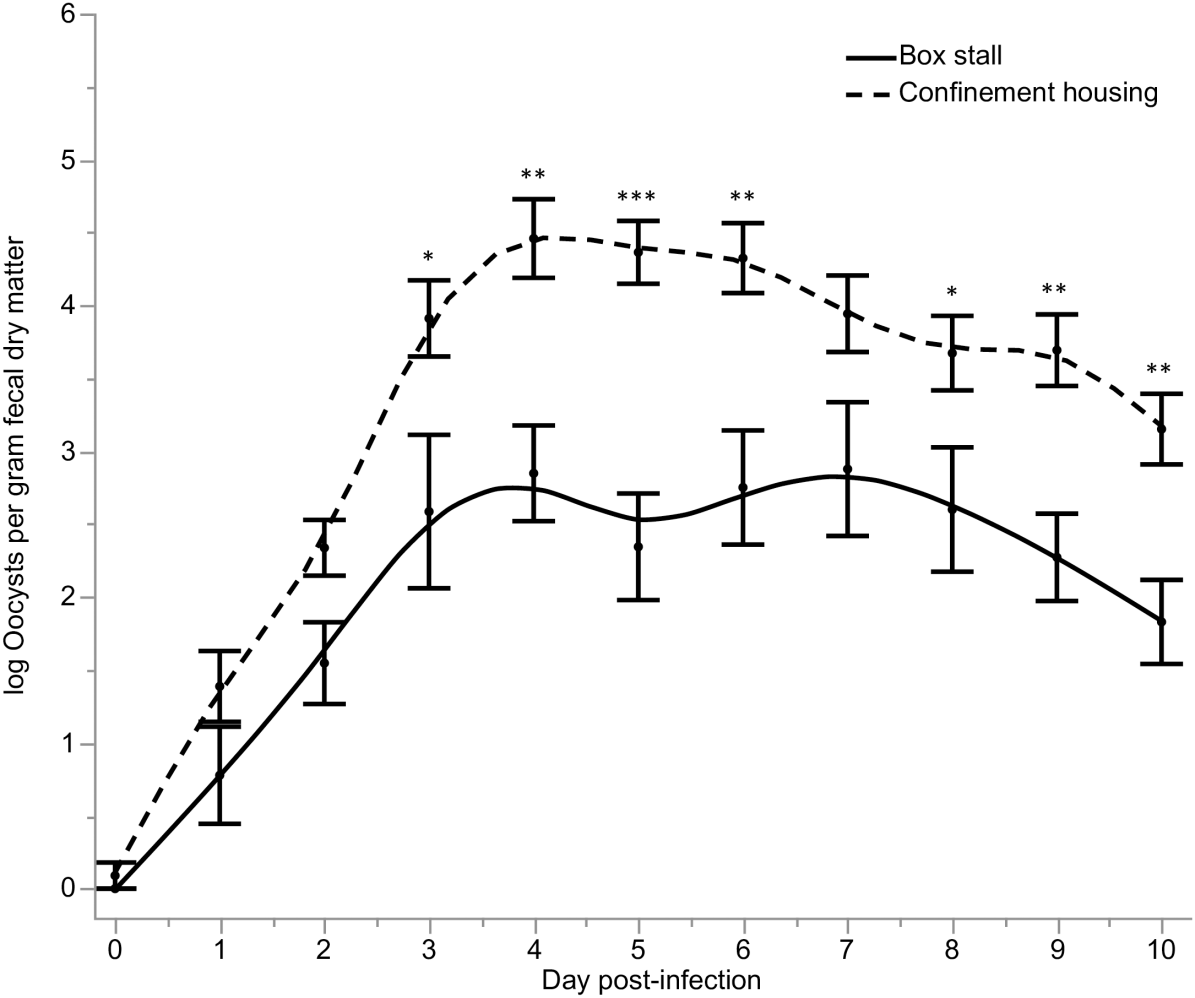
Comparison (mean ± SE) of log oocysts enumerated per gram of fecal dry matter in confinement housing (CH) (n = 12) and box stall (BXS) (n = 9) calves experimentally infected with *C*. *parvum*. * P < 0.05. ** P < 0.006. *** P = 0.0001.

When comparing across calves in confinement housing (n = 12) and calves in box stalls (n = 9), calves in confinement housing shed significantly more oocysts in their stool (*P* = 0.05) (Fig 2). The mean peak in oocyst shedding for confinement housing calves was log 7.5 oocysts/gram fecal dry matter, which is nearly 3 orders of magnitude greater than the mean peak in box stall calves. Fecal dry matter percentage was significantly lower in CFC samples from confinement housing calves as compared to IC samples from box stall calves (*P* = 0.0003), however, there was no significant difference (*P* = 0.13) in fecal dry matter percentage for IC samples collected from confinement housing and box stall calves (Tables 1 and 2).

**Table 2:** Descriptive summary of study end-points for calves in confinement housing (n = 12) and box stalls (n = 9) from days 0 to 10 post-infection.

Parameter		Confinement Housing	Box Stall Housing
Serum cortisol (ng/mL)	mean ± SD	53.8 ± 30.5	30.1 ± 19.6*
	IQR	29.1–70.9	13.0–43.7
Daily weight gain (kg)	mean ± SD	-0.1 ± 0.2	-0.1 ± 0.3
	IQR	-0.2 –-0.04	-0.3–0.2
Daily milk replacer consumption (%)	mean ± SD	73.4 ± 29.4	89.8 ± 16.2*
	IQR	50.4–100	86.2–100
Daily fecal dry matter percentage^a^	mean ± SD	9.9 ± 9.6	13.3 ± 9.2*
	IQR	3.8–11.7	6.8–17.1
Daily log oocysts/g fecal dry matter^a^	mean ± SD	3.1 ± 2.0	2.6 ± 1.3*
	IQR	1.8–4.4	2.2–3.5

*P–value < 0.02

^*a*^ Stool data for confinement housing and box stalls calves, was collected via complete fecal collection and interval collection, respectively.

For days 0–10 post-infection, calves in confinement housing experienced significantly more severe clinical outcomes in comparison to box stall calves across all study end-points except for average daily weight gain, even though calves in confinement housing consumed less milk replacer (*P* = 0.0007) (Table 2). Calves in confinement housing required significantly more fluid therapy to maintain their hydration status both during the 10 day period of confinement (*P* = 0.009) and after being removed from confinement and placed in box stalls (*P* = 0.01) (Table 3) (S2 Fig). On average, for days 0–10 post-infection, confinement housing calves required 3.5 L/day of parenteral or oral fluid therapy while box stall calves required 0.25 L/day (Table 3). Calves in confinement housing were also more likely to become injured. The frequency of abrasions or lacerations was significantly greater (*P* = 0.002) in confinement housing calves as was the frequency of pressure sores (*P* = 0.03) (Table 4). Confinement housing calves received significantly more non-fluid therapy supportive care (*P* = 0.01) in the form of administered pain medication, topical eye and skin treatments, wound care, and bandage changes.

**Table 3:** Descriptive summary of supportive care administered to calves in confinement housing (n = 12) and box stalls (n = 9).

Medical Intervention		Confinement Housing	Box Stall
Daily volume of fluids administered (L)^b^ *0–10 days post-infection*	mean ± SD	3.5 ± 3.9	0.25 ± 0.7*
	median	2.8	0
	range	0–12.6	0–2
Daily volume of fluids administered (L) ^b^ *Entire enrollment period*	mean ± SD	3.1 ± 3.3	0.4 ± 0.7*
	median	3	0.75
	range	0–12.6	0–2
Daily Non-Fluid Treatments ^c^ *Entire enrollment period*	mean ± SD	7.8 ± 17.9	0*
	median	1	0
	range	0–63	0

*P–value < 0.01

^*b*^ The daily fluid volume reported includes parenteral fluids and oral electrolytes.

^*c*^ Non-fluid treatments includes administration of pain medication, topical eye ointment for prevention or treatment of corneal ulcers, provision of topical skin medication for treatment of abrasions and lacerations, and bandage changes for the management of pressure sores.

**Table 4:** Type and frequency of injury experienced by calves in confinement housing (CH) (n = 12) and box stalls (BXS) (n = 9) over the entire period of enrollment.

Injury	Frequency		P–value
	CH	BXS	
Abrasion or Laceration	71%	0	0.002*
Pressure Sore	50%	0	0.03*
Lameness	17%	0	0.4
Corneal Ulcer	15%	0	0.5

* *P* < 0.05

**Fig 3:**
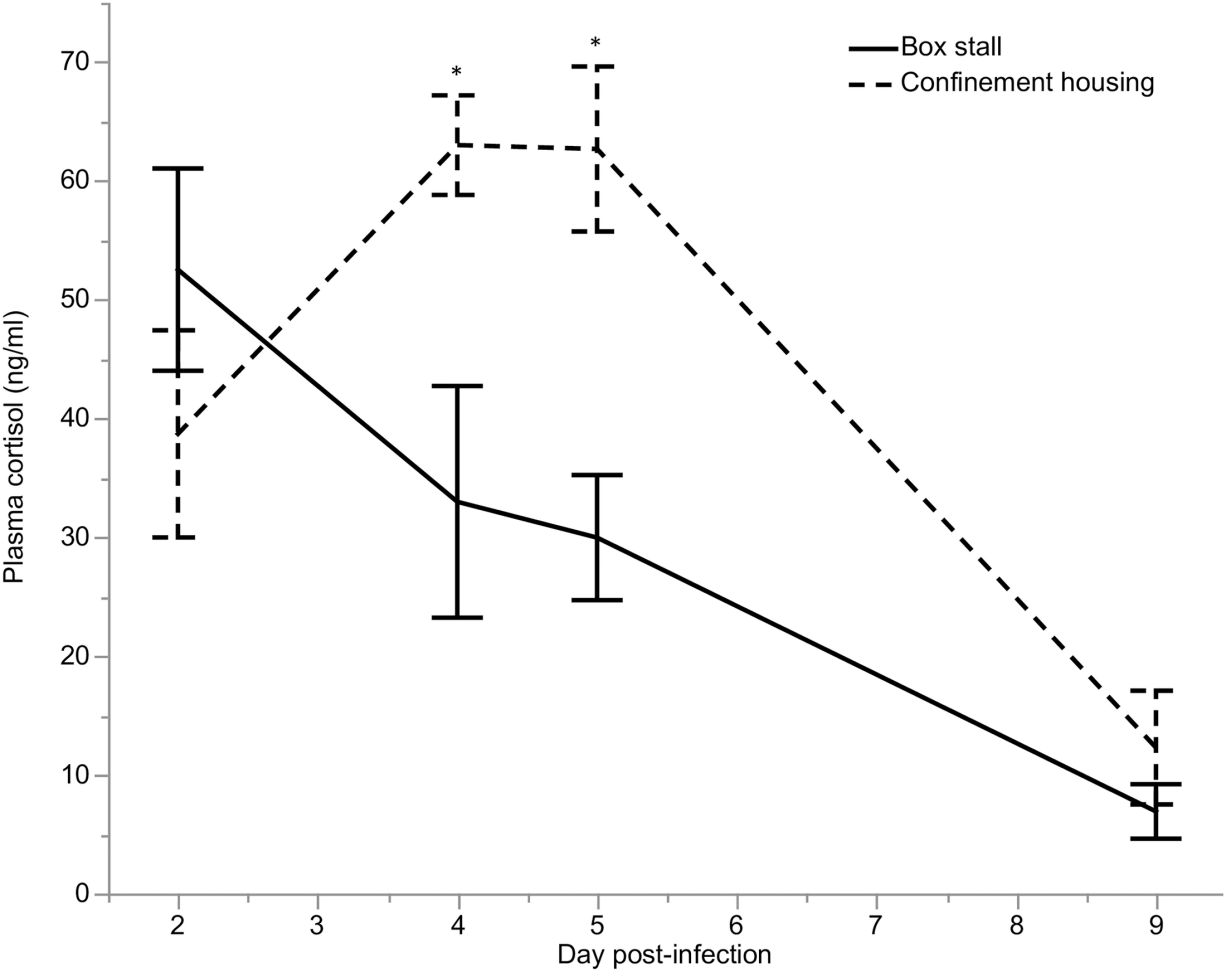
Comparison of plasma cortisol levels in confinement housing (CH) (n = 10) and box stall (BXS) calves (n = 5) experimentally infected with *C*. *parvum*. * P < 0.05.

**Fig 4:**
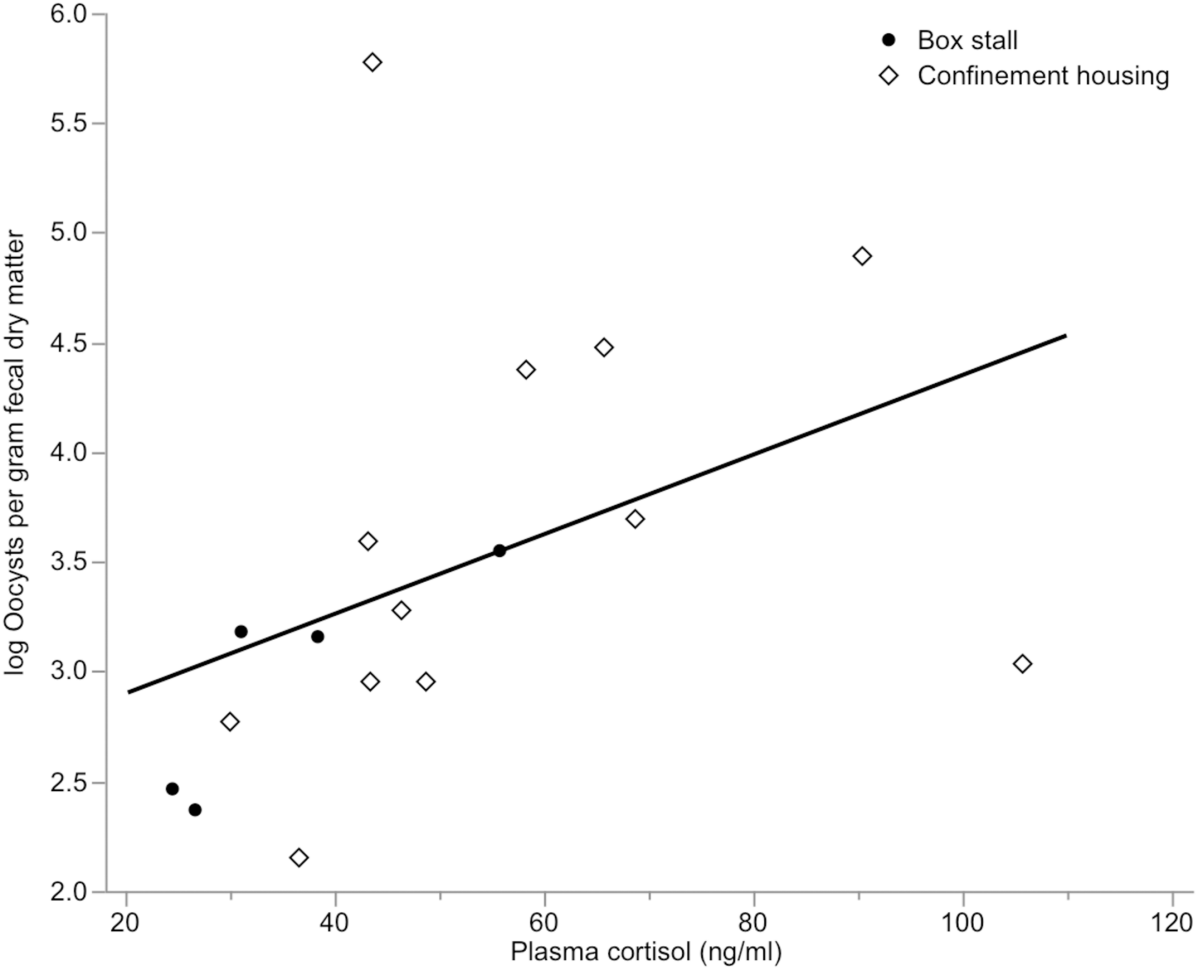
Mean plasma cortisol level and severity of fecal oocyst shedding in confinement housing (n = 10) and box stall calves (n = 5) experimentally infected with *C*. *parvum*.

Plasma cortisol samples from infected confinement housing calves (n = 10) and box stall calves (n = 5) were evaluated on days 2, 4, 5, and 9 post-infection. The distributions of plasma cortisol and log oocysts per gram of fecal dry matter were plotted and 2 outliers in the confinement housing group were identified and removed from analysis. The first measured plasma cortisol value for each of these calves was greater than 110 ng/ml. Testing was limited to 10 calves in confinement and 5 calves in box stalls due to sample loss. Mean plasma cortisol levels were significantly higher in confinement housing calves (53.6 ± 31 ng/ml) than in box stall calves (32.2 ± 21 ng/ml) (*P* = 0.002) (Fig 3). As the mean plasma cortisol level increased, the mean log oocysts per gram of fecal dry matter significantly increased (*P* < 0.0001) (Fig 4) (Table 5). For every 25 point increase in plasma cortisol there was a 1.0 log increase in oocyst shedding (Fig 4). Both housing groups initially had similar plasma cortisol levels, but the confinement housing calves experienced significant increases on days 4 and 5 post-infection (*P* = 0.01 and *P* = 0.03, respectively). This coincides with the peak in fecal oocyst shedding.

**Table 5:** Bivariate analysis evaluating the relationship between plasma cortisol levels and log oocysts per gram of fecal dry matter in calves challenged with *C*. *parvum* (n = 17).

Explanatory Variable	Outcome: log Oocysts per gram of fecal dry matter		
	Estimate	95% CI	P—value
Intercept	1.7	0.7–2.9	< 0.0002*
Plasma cortisol (ng/ml)	0.04	0.01–0.06	< 0.0001*

* *P* < 0.05

To determine the best predictors of total fecal output, backward stepwise regression was carried out. The following explanatory variables were entered into the model: plasma cortisol, percent of milk replacer consumed, log oocysts per gram of fecal dry matter, and fecal dry matter percentage. Also included were the interactions for log oocysts per gram of fecal dry matter and plasma cortisol, and for log oocysts per gram of fecal dry matter and fecal dry matter percentage. The interaction terms were found to be non-significant and were removed from the model. The remaining variables were retained. Log oocysts per gram of fecal dry matter was found to be non-significant (*P* = 0.3) and was removed. The best predictors of total fecal output were plasma cortisol (*P* = 0.02), percent of milk replacer consumed (*P* = 0.03), and fecal dry matter percentage (*P* = 0.01) (Table 6).

**Table 6:** Regression analysis to determine the best predictors of total fecal output (kg) in confinement housing calves (n = 21).

Explanatory Variable	Outcome: Total fecal output (kg)		
	Estimate	95% CI	P—value
Intercept	-4.9	-15.6–5.8	0.4
Plasma cortisol (ng/ml)	0.1	0.02–0.18	0.02*
Percent of milk replacer consumed	0.09	0.01–0.17	0.03*
Fecal dry matter percentage	-0.2	-0.4 –-0.06	0.01*

* *P* < 0.05

## Discussion

Recent large multicenter studies investigating etiologies of diarrhea in children have demonstrated that *Cryptosporidium* spp. is one of the main pathogens contributing to the global burden of pediatric diarrhea, yet there are no vaccines to prevent infection and the only licensed drug for treatment has poor efficacy [7, 8]. Efforts to accelerate identification of a lead drug candidate have generated increased demand on the calf model of cryptosporidiosis. This surge in demand has underscored the need to critically evaluate and refine the calf model of cryptosporidiosis in order to garner the best data possible for application in veterinary medicine and translation to human patients. Alarmingly, as the model has been more frequently applied, inconsistencies in model execution have come to light. One of the most pronounced inconsistencies pertains to use of confinement housing for research calves. The desire to mimic human challenge studies and collect the total fecal output from research calves has necessitated the use of confinement housing [30, 31, 38, 39]. Use of confinement housing conflicts with The Five Freedoms of Animals, which are commonly used to define parameters for animal welfare and to determine if animal housing, environment, and handling are adequate [40–42]. In the case of confinement housing, the freedom to express natural behaviors is not observed. In research settings, the five freedoms may be compromised if there is scientific justification or if no other acceptable alternative exists. To date, no one has evaluated confinement housing side-by-side with box stall housing to determine if box stall housing is an acceptable alternative in the calf model of cryptosporidiosis. Our study demonstrates that use of confinement housing is unnecessary to measure study end-points such as total oocyst excretion and diarrhea quantity and quality. Moreover, the use of confinement housing appears to stress calves and lead to increased oocyst excretion that may confound data and underestimate the efficacy of the chemotherapeutics being studied.

Our findings challenge the assumption that CFC is required to get accurate measurements of oocyst excretion or the quantity and quality of diarrhea. First, we did not find a difference in log fecal oocysts enumerated per gram of fecal dry matter in CFC or IC samples among calves in confinement. Previously, it was thought that homogenization of the CFC sample would result in more accurate oocyst enumeration [25], but this was not supported by our data. To the contrary, there was greater variation in oocyst enumeration for CFC samples, as is evidenced by the larger standard error bars (Fig 1). Since these counts are standardized to the fecal dry matter percentage, this would indicate that these differences are attributable to increased water content of the stool sample. It has also been suggested that CFC must be collected and homogenized due to possible diurnal variations in fecal oocyst shedding [25, 26], but we observed no significant difference in the number of oocysts enumerated in samples collected in the morning or evening.

It is important to note that while CFC permits capture of the total fecal output, the entire CFC sample is not analyzed. For both CFC and IC a 200 mg aliquot of stool is homogenized with equal amounts of PBS. After qPCR is performed, oocyst counts are standardized by fecal dry matter percentage. Therefore, our findings indicate that a representative stool sample can be attained via IC and collection of the total fecal output is not necessary in order to attain a representative stool sample for oocyst enumeration via qPCR.

The fecal dry matter percentage was significantly lower in CFC samples as compared to IC collected from calves in confinement. This was controlled for in oocyst enumeration by standardizing to the fecal dry matter percentage. The increased water content in CFC samples could be from more severe diarrhea or improved ability to measure water content due to sample homogenization. Alternately, it could be due to urine contamination of the fecal bin which has been previously described or to increased urine production secondary to increased frequency of fluid therapy [25]. Although adult diapers were secured to the calves using Duct Tape (3M, St. Paul, MN) and Elastikon (Johnson & Johnson, New Brunswick, NJ) diapers did slide out of place with calf movement and exposed the penis. In addition, during the first 4–5 days of confinement, calves were small enough to turn around in the stall, thus there were instances when they defecated in the urine bin, which would falsely lower the fecal dry matter percentage of stool collected from the fecal bin and falsely elevate the volume of urine collected (S1 Fig). If the lower fecal dry matter percentage detected in confinement housing calves is a true finding, indicating that CFC better detects increased water content in the stool, this would indicate that confinement exacerbates severity of diarrhea. This is a possibility, given that calves in confinement housing required significantly more supportive care than calves in box stalls. Calves in confinement housing received an average of 3.5 L of fluid therapy each day, as compared to box stall calves, which only received 0.25 L/day. Thus, confinement housing calves may experience greater volume of fluid elimination due to greater volume of fluid support. However, when comparing IC samples from confinement housing calves to IC samples from box stall calves, there is no significant difference in fecal dry matter percentage. Thus, it is probable that the difference in fecal dry matter percentage between CFC and IC samples for confinement housing calves is at least partially attributable to urine contamination of the fecal bin.

Calves in confinement housing shed significantly more oocysts in their stool. At peak shedding, this difference was 2 orders of magnitude greater in confinement housing calves. Since there was no difference in shedding detected in IC or CFC samples from confinement housing calves, it stands to reason that the difference in shedding detected between box stall and confinement housing calves is a true effect. Moreover, since all qPCR data was standardized to the fecal dry matter percentage, this data is not susceptible to dilutional effects of increased water content in the stool sample. It is possible that this difference in fecal oocyst shedding can be explained by the significant difference in plasma cortisol concentration for confinement housing and box stall calves. In healthy calves, plasma cortisol concentration is reported to be high at birth (40–50 ng/ml) and gradually decreases over the first 20 days of life (15–20 ng/ml) [34, 43], which is consistent with our findings for box stall calves. Calves in confinement housing did not follow this pattern. Plasma cortisol was elevated at 4–5 days post-infection (62 ng/ml) in confinement housing calves, coinciding with the observed peak in fecal oocyst shedding. Thus, it is feasible that increased stress due to confinement exacerbated the degree of fecal oocyst shedding in confinement housing calves. This conclusion is supported in the bivariate analysis in which plasma cortisol level and log oocysts per gram fecal dry matter were significantly associated. It could be argued that confinement housing effectively creates an immunosuppressed calf model of cryptosporidiosis. This may be deemed desirable by *Cryptosporidium* researchers, as many children infected with *Cryptosporidium* are immunocompromised due to other comorbidities. However, unlike immune suppressed mouse models, where diminished immune function is intentionally induced, the presumed reduction in immune function due to hypercortisolemia for calves in confinement housing is a pathological state. While chemotherapeutic efficacy in the face of immunocompromise is desirable, when testing chemotherapeutics in animal models, the degree of immune suppression induced must be predictable and repeatable.

Our findings also indicate that calves in confinement housing were substantially more physically and metabolically decompensated than calves in box stalls. Confinement housing calves required more fluid therapy and ate significantly less milk replacer. Reduced milk replacer consumption is an indication of reduced calf wellbeing. In the calf model of cryptosporidiosis this has additional relevance, as failure to meet daily fluid requirements through consumption of milk replacer puts calves at greater risk for severe dehydration and development of metabolic acidosis, which can result in calf death or removal from the study. Confinement housing calves also received significantly more non-fluid treatments. Not only does this indicate reduced calf welfare, it also indicates increased time demands on study personnel for provision of care to sick calves. Box stall calves experienced no abrasions, lacerations, or pressure sores. These types of injuries only occurred in confinement housing calves, as they were more likely to injure themselves on the metal framework of the calf stall, and the lack of shock absorption when lying down predisposed calves to repeated injury over their joints and bony prominences. The severity and duration of these injuries may have been impacted by elevated plasma cortisol levels. Mice placed in confinement to induce repeated restraint stress experienced impaired wound healing, partially through stress-induced glucocorticoid release [44].

Given these findings, we wanted to determine if variables that could be collected without the use of confinement housing would accurately predict the total fecal output produced by a calf experimentally infected with *C*. *parvum*. In our regression model, holding other variables constant, plasma cortisol, the percent of milk replacer consumed, and the fecal dry matter percentage from IC samples were the best predictors of the total fecal output measured via CFC. These variables accounted for the greatest amount of inter-calf variation in total fecal output. This indicates that when used in combination, these study end-points are better measures of a treatment effect than total fecal output alone. The regression model also suggests that the total fecal output is sensitive to hypercortisolemia. Interestingly, the log oocysts per gram of fecal dry matter was not a good predictor of total fecal output. A previous study has shown that the magnitude of oocyst shedding tracks with the ordinal measures of severity of diarrhea [36]. When considered alongside our data, this indicates that ordinal measures of severity of diarrhea and total fecal output are not synonymous, and that fecal dry matter percentage is a better proxy measure of total fecal output.

The findings of this study suggest that confinement housing of calves may affect study end- points that are important for the evaluation of novel chemotherapeutics targeting *Cryptosporidium*. Fecal oocyst shedding, appetite, and frequency of fluid therapy are study end-points that were all significantly impacted by the use of confinement housing. Our results indicate that representative data can be captured via IC from calves housed in box stalls, and that this data is less likely to be impacted by the immunomodulatory effects of stress hypercortisolemia. While collection of total fecal output mirrors methodologies used in human challenge studies, use of confinement to capture total fecal output confounds data used to assess other study end-points and reduces overall comparability to the human challenge model. Given that confinement housing calves experience increased severity of illness, higher plasma cortisol levels, greater risk of injury, and higher requirements for fluid therapy, use of confinement housing to evaluate novel chemotherapeutics targeting *Cryptosporidium* spp. may result in reduced ability to detect a treatment effect.

That said, our study is limited by the fact that we did not compare outcomes in the two housing systems during a trial of an actual *Cryptosporidium* therapeutic. However, box stall housing has recently been used successfully in a trial evaluating a novel drug targeting *Cryptosporidium* [45]. Our study was also subject to potential bias due to the inability to blind observers to housing system. For this reason, we did not report and evaluate subjective observer assessments in this study, such as mentation. These assessments were restricted to use in determining need for supportive care. To reduce risk of bias in evaluation of injuries, severity was not assessed. Instead assessments of injuries, such as abrasion or pressure sore, were dichotomous: present or not present. Blinding was maintained for laboratory analysis of stool and blood samples. While we were able to report an association between plasma cortisol and oocyst shedding, this study was not designed to assess stress as a primary end-point. Cortisol is just one marker of stress. A more complete assessment of stress, including behavioral assessment, and other markers such as substance P, would aid in the interpretation of the data. Another concern is the loss of calves in the confinement group due to death, which could result in imprecise estimates. However, we feel that imprecise estimates are the minor concern relative to the major concern of compromised research animal welfare. When considered together, the risk of imprecise measurements due to loss as well as reduced welfare resulting in death and injury, further substantiates the need to revise the methods used for studying cryptosporidiosis in the calf model. Lastly, while the study authors have extensive experience using box stalls, this was our first study using confinement housing. It could be argued that in more experienced hands the study findings may have been different. To address this, we consulted with researchers currently executing these studies in confinement and followed protocols identical to those previously published [25].

In conclusion, it is our opinion that box stall housing should be preferentially used in the calf model of cryptosporidiosis, as confinement housing compromises calf welfare, alters important outcomes, appears to stress calves, and leads to sicker and more heavily infected calves.

## Supporting information

S1 Table Rubric for the evaluation of appetite, mentation, fecal consistency, and hydration. (DOCX)

S1 Fig Photograph of enrolled calves in confinement housing. (TIF)

S2 Fig Fluid therapy flow chart. Calculation of calf fluid deficit and volume to administer to maintain 5% dehydration.
(TIF)
