# Supplementary material for: Impact of confinement housing on study end-points in the calf model of cryptosporidiosis
Source: PLoS Negl Trop Dis. 2018 Apr 25;12(4):e0006295. doi: 10.1371/journal.pntd.0006295 (PMC5937795; doi:10.1371/journal.pntd.0006295)
Supplement: S1 Table — (DOCX) [file pntd.0006295.s001.docx]

|  | **Appetite** | **Mentation** | **Fecal Consistency** | **Hydration** |
| --- | --- | --- | --- | --- |
| **1** | **Normal.** Calf may need some coaxing at first. Calf will eat vigorously, often twitching its tail as it eats. | **Normal.** Calf is alert and watches the caretakers, reacts when caretakers enter the stall. It may rise to meet them. | **Normal**. Feces retain form. The feces may be sticky but do not flow across a surface. | **Normal to mild dehydration (< 5%).** Calf is euhydrated with no clinical signs. Or, calf has diarrhea (fecal score ≤ 3) but no other clinical signs (appetite & mentation score = 1). |
| **2** | **Mild to moderate inappetence.** Calf eats with some coaxing, but not aggressively. Suckle reflex is present but may not be vigorous. | **Mild to moderate depression.** Calf acknowledges caretakers, but is reluctant to rise without substantial coaxing. | **Mild diarrhea**. Forms a patty. Slight water content which may or may not slowly flow across a surface. Will leave some material adherent to a surface. | **Moderate (6-8%) dehydration.**  Fecal score ≥ 2 with a skin tent test lasting 2-6 seconds. Mucous membranes may be tacky. Mild to moderate enophthalmos. Mentation score ≥ 2. Appetite score ≥ 2. |
| **3** | **Severe inappetence.** Calf refuses to eat even when coaxed. Suckle reflex is weak or absent completely. | **Severely Depressed.** Calf pays no attention to caretakers, and will not rise even with coaxing. Calf may or may not be moribund. | **Moderate-to -severe diarrhea.** Forms a puddle. Sufficient water content to flow across a surface leaving little to no adherent material. Calf may have very watery feces followed by formed stool and still be scored a 3. | **Severe (> 8%) dehydration.** Fecal score ≥ 2. Skin tent test lasting > 6 seconds. Mucous membranes may be dry. Severe enophthalmos, eyelashes may be touching globe. Mentation score ≥ 2. Appetite score = 3. |
