# Supplementary figures and images for: Impact of confinement housing on study end-points in the calf model of cryptosporidiosis
Source: PLoS Negl Trop Dis. 2018 Apr 25;12(4):e0006295. doi: 10.1371/journal.pntd.0006295 (PMC5937795; doi:10.1371/journal.pntd.0006295)

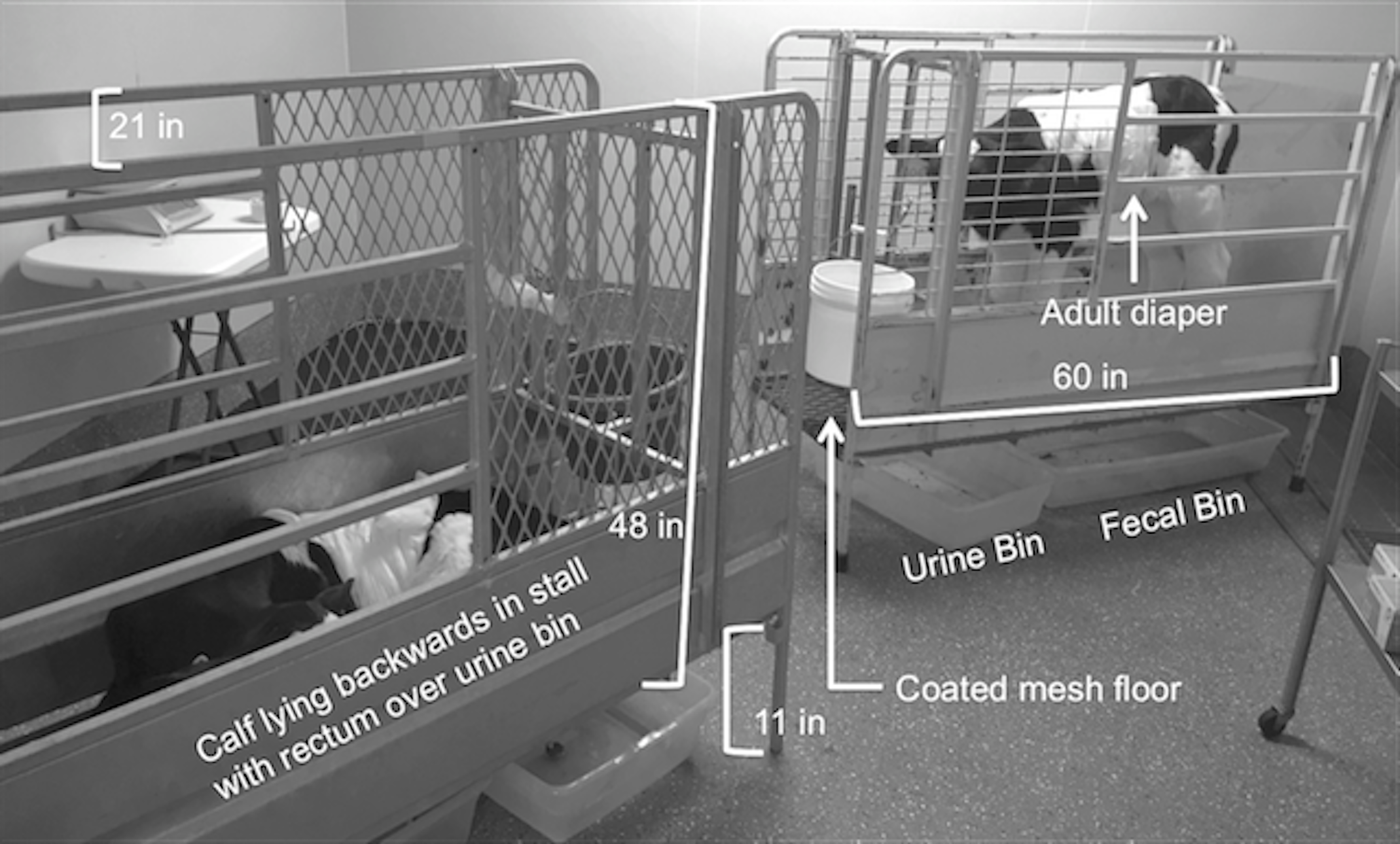

Supplement: S1 Fig — (TIF) [file pntd.0006295.s002.tif]

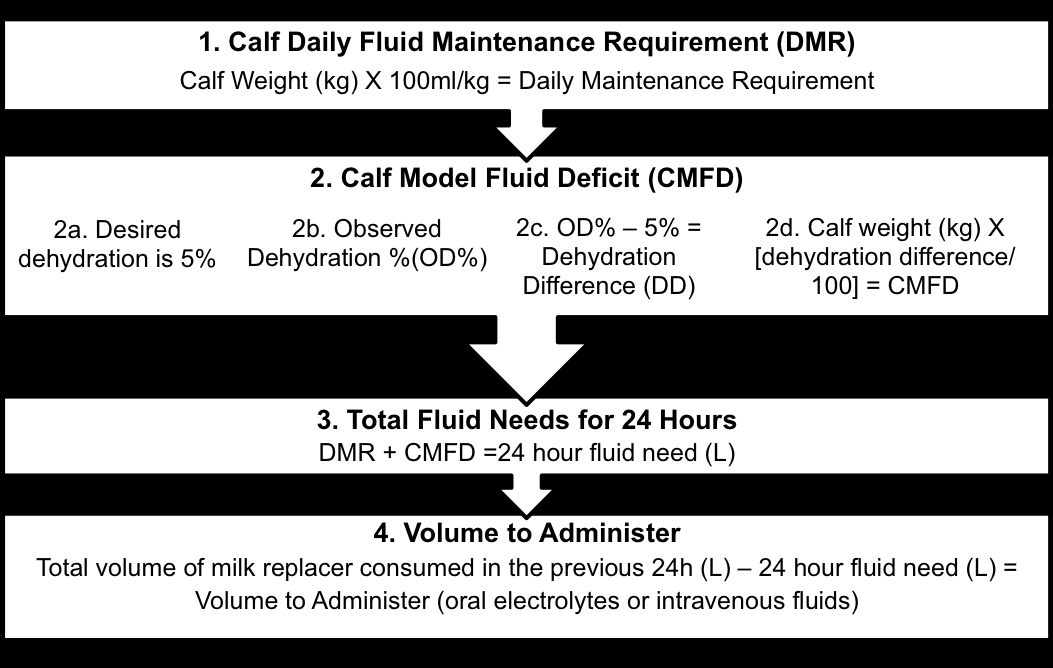

Supplement: S2 Fig — Calculation of calf fluid deficit and volume to administer to maintain 5% dehydration. (TIF) [file pntd.0006295.s003.tif]
